# DNA Copy Number Variations as Markers of Mutagenic Impact

**DOI:** 10.3390/ijms20194723

**Published:** 2019-09-24

**Authors:** Galina Hovhannisyan, Tigran Harutyunyan, Rouben Aroutiounian, Thomas Liehr

**Affiliations:** 1 Department of Genetics and Cytology, Yerevan State University, Alex Manoogian 1, 0025 Yerevan, Armenia; galinahovhannisyan@ysu.am (G.H.); tigranharutyunyan@ysu.am (T.H.); genetik@ysu.am (R.A.); 2 Institute of Human Genetics, Jena University Hospital, Friedrich Schiller University, Am Klinikum 1, D-07747 Jena, Germany

**Keywords:** copy number variation, human, animal and plant cells, chemical mutagens, radiation

## Abstract

DNA copy number variation (CNV) occurs due to deletion or duplication of DNA segments resulting in a different number of copies of a specific DNA-stretch on homologous chromosomes. Implications of CNVs in evolution and development of different diseases have been demonstrated although contribution of environmental factors, such as mutagens, in the origin of CNVs, is poorly understood. In this review, we summarize current knowledge about mutagen-induced CNVs in human, animal and plant cells. Differences in CNV frequencies induced by radiation and chemical mutagens, distribution of CNVs in the genome, as well as adaptive effects in plants, are discussed. Currently available information concerning impact of mutagens in induction of CNVs in germ cells is presented. Moreover, the potential of CNVs as a new endpoint in mutagenicity test-systems is discussed.

## 1. Introduction

Iafrate et al. and Sebat et al. first described euchromatic large-scale copy number polymorphisms in the human genome [1,2]. In recent decades, copy number variation (CNV) has been recognized as the common type of polymorphism in the genomes of humans, animals and plants [3,4,5,6]. CNVs result from unbalanced DNA rearrangements that increase or decrease the DNA content leading to changes in the number of copies of a particular DNA sequence. Hence CNVs are structural changes within chromosomes and distinguished from whole chromosome gains and losses as latter are a separate class of cytogenetic alterations (= aneuploidy). Typically, CNVs encompass relatively large DNA segments (from 50 bp to several Mb) [5,6], whereas smaller elements are known as insertions or deletions (indels) variants [5]. Currently, it is estimated that common CNVs occur in approximately 9.5% of the human reference genome and have non-random distribution, however, they frequently occur in replication origins and palindromic regions [5].

By origin, CNVs can be classified as de novo (occurring spontaneously) or inherited. Parental germ cell CNVs can be detected in all cells of the offspring while de novo CNVs are found in particular populations of cells (e.g., in tumor before and/or after therapy). Copy number alterations or aberrations (CNAs) are changes in genomic copy number of cells and are referred to by different terms depending on their size [7,8,9]. Since CNVs and CNAs are sometimes used as synonyms, to avoid confusions in this review, changes in DNA copy number of any origin occurring within chromosomes will be referred to as CNVs.

There are two major classes of CNVs: recurrent and non-recurrent. A major mechanism leading to recurrent CNV is meiotic unequal crossing over, or non-allelic homologous recombination, mediated by flanking repeated sequences or segmental duplications. Due to breakpoints cluster within defined regions, the extent of recurrent CNVs is essentially identical, even in unrelated individuals. In contrast, non-recurrent CNVs have breakpoints that generally lie within unique sequence and can arise by several different mechanisms, including non-homologous end joining and or aberrant replication, suggesting a mitotic origin [10].

CNVs are an important source of both normal and pathogenic variations in the human genome. Pathogenic CNVs are typically very large and contain multiple genes, involved in development and genes that have high evolutionary copy number conservation across mammals. Benign CNVs are often small, intergenic, or encompass genes that can tolerate a change in copy numbers [11]. Interpreting the pathogenicity of CNVs remains challenging, nevertheless, abnormal phenotypes in up to 20% of individuals can be explained by CNVs [6].

CNVs can affect gene expression by simple gene dosage effects, and can include duplication or deletion of gene regulatory regions and alterations in physical proximity of genes and regulatory elements. A better understanding of the effects of CNVs on gene expression is needed to estimate the role of genetic variation in complex traits [12]. In cancer cells, later stabilized gain type of CNVs occur in oncogenes, in which the deletion type of CNVs is stabilized when tumor suppressor genes are affected.

The detection of large cytogenetically visible CNVs (> 5Mb) was accomplished by karyotyping for 50 years [13]. Rapid advancement in the technology of analysis of the human genome created new possibilities for CNV investigation. Two primary technologies for the detection of CNVs across the whole genome are array comparative genomic hybridization (aCGH) and the recently introduced next-generation sequencing (NGS) [5,6].

Iafrate et al. had introduced the idea of a public CNV database [1]. Now the Database of Genomic Variants provides a publicly accessible, comprehensive curated catalogue of CNVs and structural variations that are found in the genomes of control individuals from worldwide populations [14,15]. A high-resolution map of human benign CNVs among healthy individuals of various ethnicities was developed by Zarrei et al. [5]. 

CNVs are important in genome variation and genetic disease, with new mutations arising frequently in the germline and somatic cells. CNV formation, like all classes of mutation, will likely include exposure to environmental mutagens and inherited genetic predispositions. The spectrum of DNA damaging agents known to lead to increased rates of CNV formation is still quite limited [10,16]. In this review, we will focus on radiation- and chemical mutagen-induced CNVs in cells, organisms and germline. Particular attention will be paid to CNV formation during adaptation to the environment and mutation breeding. We will also discuss the results of our studies of CNVs induced by aflatoxin B1 and accelerated electrons.

## 2. Radiation-Induced CNVs In Vitro

Ionizing radiation (IR) is a mutagen that promotes tumorigenesis. One severe consequence of IR is the development of secondary malignant neoplasms, a radiotherapy-associated complication in survivors of cancers [17]. Research in the area of radiation-induced genomic instability and its implications for radiation carcinogenesis is highly actual; especially because radiation-induced CNVs in germline can be transmitted to the next generation [18]. 

The first genome-wide copy number assessment of low-dose gamma radiation-induced genomic instability in vitro was realized in human lymphoblastoid TK6 cell line using aCGH [19]. Irradiated TK6 cells displayed patterns of instability similar to those seen in radiation-induced tumors. Particularly gains with the highest densities in 3q, 13q and 20q and losses in the single X-chromosome were recognized. The observed abundance of DNA gains in TK6 cells suggest that chronic low-dose exposures have the potential to produce genomic changes at pre-neoplastic stage that later can be propagated in tumors.

Genomic lesions induced by acute exposure of A549 lung adenocarcinoma cells to X-rays were studied by aCGH and fluorescence in situ hybridization (FISH). FISH revealed radiation-induced specific translocations and aneuploidies. aCGH revealed distinct and recurrent mutations, overrepresented by deletions. Moreover, fragile sites FRA3B and FRA16D were shown to be targets of radiation-induced mutations [20].

IR effectively induces de novo CNV mutations in cultured normal human fibroblasts [21]. These IR-induced CNVs are found throughout the genome, with the same hotspot regions seen earlier after aphidicoline and hydroxyurea-induced replication stress [22,23]. The similarity to aphidicoline/hydroxyurea-induced CNVs suggests that low-dose IR induces CNV through a replication-dependent mechanism. IR-induced DNA strand breaks can collapse the replication fork and result in giving rise to CNVs [21].

More than half of the X-irradiated clones of primary human fibroblasts displayed an increased rate of CNVs and loss of Y-chromosome compared to controls. CNV breakpoints were clustered in specific chromosomal regions, in particular 3p14.2 and 7q11.21, coinciding with common fragile sites. Both genetic and epigenetic changes occur in the progeny of exposed cells that were not damaged directly by radiation, likely contributing to radiation-induced carcinogenesis [24].

We studied CNVs in chromosomal regions 1p31.1, 7q11.22, 9q21.3, 10q21.1 and 16q23.1 in cultured normal human blood leukocytes irradiated with laser-driven electron bunches using parental origin determination FISH (POD-FISH) [25]. Irradiation of cells significantly increased levels of duplication in all analyzed chromosomal regions earlier reported to be sensitive to ionizing radiation. Our data confirmed that hotspots of de novo CNV mutations defined in normal human fibroblast cell lines after ionizing radiation [21] also represent targets for accelerated electrons.

## 3. Chemical Mutagen-Induced CNVs In Vitro

Exposure to environmental chemicals may contribute to the formation of CNV and this influences disease onset and pathogenesis; however, studies on this topic are still in their infancy.

The first genome-wide study of chemical mutagen-induced CNV was realized in human cells treated by classical replication inhibitor aphidicoline. CNVs were identified using aCGH [22]. Aphidicolin-induced replication stress in normal human fibroblast cell line HGMDFN090 leads to a high frequency of CNVs of tens to thousands of kb across the human genome that closely resemble many normal and pathogenic human CNVs. Treatment with aphidicolin results in the induction of submicroscopic deletions and duplications from 25 kb to several Mb in size. This earlier unrecognized consequence of replication stress suggests that replication fork delay and subsequent error-prone repair are important mechanisms in the formation of CNVs (including pathogenic) in humans. To expand the data on aphidicolin-induced CNVs obtained by aCGH [22], more sensitive to small variants, high-resolution single-nucleotide polymorphism (SNP) arrays and mate-pair sequencing were applied in the same human cells [26]. The results confirmed that aphidicolin stimulates formation of CNVs that closely resemble human pathogenic CNVs and the subset of larger non-homologous constitutional CNVs. Relative to the constitutional CNVs, aphidicolin-induced CNVs showed a much larger median size of 148 kb, while most constitutional CNVs are <10 kb.

To know precisely whether CNV induction could be attributed to replication stress or was specific to aphidicolin-mediated DNA polymerase inhibition [22,26], the effects of therapeutic doses of clinically relevant replication inhibitor hydroxyurea (HU) on CNV formation were studied [23]. Treatment of human fibroblast with an HU-induced high rate of CNVs overlapped with the size and breakpoints of pathogenic CNVs and those induced by aphidicolin. Analysis of localizations of de novo CNVs revealed clusters on different chromosome loci. The average size of deletions and duplications induced by HU was 132 kb (1 kb–35.7 Mb , which was slightly less than the average size (165 kb) of aphidicolin-induced CNVs (1 kb–80.3 Mb) [22]. The sizes of chemically induced CNVs are comparable with spontaneously arising CNVs in control cells, where the median size of CNVs was 187 kb (19 kb– 1.5 Mb). The authors concluded that CNVs can arise due to errors of DNA replication suggesting potential role of replication inhibitors in induction of de novoCNVs. 

The ability of ethyl methanesulfonate (EMS) and cytosine arabinoside (Ara-C) to generate CNVs was studied in a zebrafish fibroblast cell line using aCGH. Five CNVs were generated in similar genomic regions in both the EMS and Ara-C treatments indicating these regions may be more susceptible to genomic alterations in a nonchemical-specific manner. Furthermore, CNVs were correlated with altered gene expression. This study suggests that chemical exposure generates CNVs which impacts on gene expression [27].

Our research has shown that aflatoxin B1, a mycotoxin that disturbs DNA replication may also have the potential to induce de novo CNVs. With application of POD-FISH it was revealed that AFB1 induced deletions in CNVs in chromosome loci 8p21.2 and 15q11.2 in human leukocytes culture [28]. This confirms the idea of Arlt et al. [23] about the ability of inducers of replicative stress to cause changes in CNVs [23].

Table 1 summarizes comprehensive data on radiation- and chemical mutagen-induced CNVs in human and animal cells in vitro, their locations in the genome and ratio of gains and losses. It can be noted that radiation induces equally gains and losses while chemical mutagens in majority of studies induce copy number losses. It can be assumed that radiation can impact random loci of the genome while there are (presumably) genomic targets for CNV induction in case of chemical mutagen exposure in vitro.

## 4. Radiation-Induced CNVs In Vivo

Although IR is used in modern medicine, its effects are poorly characterized from a genome- or exome-wide perspective [17]. A series of studies have been undertaken to describe in vivo radiation-induced CNVs in rodents and humans. 

CNVs in spontaneous and induced by gamma radiation rat mammary carcinomas were compared using aCGH analysis. An amplification of the chromosomal region 1q12, and deletions of the chromosomal regions 3q35-q36, 5q32 and 7q11 were specific for radiation-induced carcinomas and were not observed in spontaneous forms of the disease. This was the first report, which revealed that radiation-induced mammary tumors show distinct CNV patterns [29].

Using mouse models of second malignant neoplasms, Sherborne et al. performed whole exome sequencing of IR-induced neoplasms in wild-type and tumor suppressor gene Nf1 mutant mice [17]. Overall, Nf1 mutant-derived samples showed far more copy number losses than gains compared to wild-type-derived samples (29% versus 11%), while wild-type derived tumors showed the reverse pattern, harboring more gains than losses (33% versus 8%). The authors conclude that mutational signatures found in IR-induced tumors are likely to reflect not only the DNA damage specific to the mutagen but also the influence of genetic background [17].

Swedish women treated with IR for skin haemangioma at an early age were studied for the long-term effects of IR exposure on breast cancer development [30]. In each tumor sample, 7–23 affected chromosomes were identified with CNVs occurring in one or both chromosome arms. Comparative analysis between tumors of low-dose and high-dose irradiated groups revealed 156 chromosomal regions containing in total 301 different CNVs. In the group exposed to high doses of radiation significantly larger part of the genome was affected. Higher frequencies of copy number gains were identified on chromosomes 2q, 4, 17, 21q, 22q, and copy number losses on 6q in the high-dose group. These results indicate dose-dependence of CNV frequency in breast carcinomas in the Swedish hemangioma cohort.

Zitzelsberger and Unger provided a comprehensive review of on the current knowledge of CNVs in papillary thyroid carcinoma (PTC) among victims of the Chernobyl accident [31]. Following exposure to radiation during the Chernobyl accident, PTC increased significantly in irradiated individuals. Thus, there is a strong motivation to search for novel alterations that might represent radiation-specific markers in PTC. 

Another study by Zitzelsberger et al. presented cytogenetic findings in radiation-induced post-Chernobyl childhood PTC in Belarussian patients and secondary tumors after radiotherapy [32]. Multiple structural chromosome aberrations and some novel chromosomal breakpoints of structural aberrations were more frequently detected in radiation-induced than in spontaneous thyroid tumors. A detailed analysis of one patient using aCGH revealed gains in 1p34, 1q21, 2p11.1-pter, 2q11.2-13, 3q26.2-26.3, 5q23-31, 6p21.3-pter, 7q11, 9q13-q33, 12q22-qter, 13q32-qter, 17q11.1-qter, 19, 20, X and losses in 1q42, 13q21, 15q11.1-14. Prevalence of chromosomal loci with gains over loses was observed. 

Tissue samples from 60 post-Chernobyl childhood thyroid tumors have been further investigated by aCGH. Chromosomal imbalances were found in 30% of tumors. The most frequent DNA copy number changes involved chromosomes 2, 7q11.2-21, 13q21-22, 21 (DNA gains), and chromosomes 16p/q, 20q, 22q (DNA losses) [33].

The prevalence of DNA gains in persons with PTC exposed in Chernobyl was shown to be two- to fourfold higher than for cases in unexposed individuals and up to 10-fold higher for the subset of recurrent gains. aCGH in 10 radiation-induced tumors of young patients demonstrated gains in 1p36.23, 1q32.1, 1q42.13, 5q31.3, 6p24.3, 6p22.2, 7p11.2, 7p14.1, 7q21.13, 7q32.1, 8p23.1, 14q32.33, 16p13.3, 16q12.2, 17q11.2, 17q25.1-25.3, 19p13.11, 19p13.43, 20q13.33, 21q22.3, 22q13.2, Xp22.33 and loss in 4q35.2 [34].

Unger et al. found the CNV pattern of 13 childhood radiation-induced PTC using aCGH method in residents living in the vicinity of Chernobyl. The authors report losses on chromosomes 1p36.31-35.3, 1p33-cen, 1q23.2-32.1, 6, 9p21.1-pter, 9q31.1, 9p23-q33.2, 13 and gains on chromosomes 19 and 21 [35].

The next study of radiation-induced PTCs from Chernobyl pediatric patients was realized with application of two high-throughput, whole-genome platforms [36]. Many regions of CNVs were detected in 10 tumors including novel regions that had never been associated with PTCs. Increases in copy numbers were consistently found on chromosomes 1p, 5p, 9q, 12q, 13q, 16p, 21q, and 22q. Deletions were observed less frequently and were mapped to 1q, 6q, 9q, 10q, 13q, 14q, 21q, and 22q. An overlay analysis of the gene expression and CNV profiles identified genes showing both CNVs and concurrent gene expression alterations. The authors concluded that CNVs also influence gene expression that probably has an effect on tumorigenesis.

According to Zitzelsberger and Unger it is an important question whether CNVs discriminate radiation-induced and sporadic PTC [31]. All publications reviewed made a significant contribution to the creation of a database of CNVs in radiation-induced PTCs [32,33,34,35,36]. It is obvious that a larger number of CNVs have been reported in radiation-associated compared with sporadic cases. Nevertheless, this information yet does not allow defining radiation-specific genetic markers. Thus, the issue of identifying a radiation-induced PTC-specific genetic signature is still open. Zitzelsberger and Unger consider that CNVs have a great potential to gain insights into radiation-specific changes occurring during PTC development [31]. 

In a more recent study, a signature consisting of nine CNV regions located on chromosomal bands 1p21.1, 2q35, 2q35, 6p22.2, 7q11.22-11.23, 7q21.3, 16q24.3, 17q21.31, 20p11.23-11.21 was characterized in Chernobyl clean-up workers and evacuees with breast cancer. The authors claim that gains and losses of cancer-related candidate genes and miRNAs observed in studied cohort have the potential to allow identification of radiation-associated breast cancer at the individual level [37]. Interestingly, gains in chromosomal region 7q11.22 were also observed in our study of radiation-induced CNVs in human leukocytes in vitro [25] indicating feasibility of CNVs in this locus for detection of radiation exposure in human cells.

The effect of A-bomb radiation exposure on genomic instability in breast cancer was studied by aCGH in archival formalin-fixed paraffin-embedded tissues. This study revealed a higher incidence of CNVs in breast cancer tissue from A-bomb survivors than in tissue from calendar year-matched control patients, suggesting a role for genomic instability during breast carcinogenesis. This was the first report of an aCGH analysis with solid tumors from A-bomb survivors [38].

Solar ultraviolet (UV) radiation generates photodimers, oxidative DNA lesions and DNA single strand breaks hindering DNA replication and transcription, as well as contributes to development of skin malignancies [39]. Replication stress can cause CNVs [10], thus it can be assumed that CNVs may accumulate in the genome as UV-induced skin tumors progress.

It is thought that growing number of cases of squamous cell carcinoma (SCC) in populations of high UV exposure regions in Australia (e.g., Queensland) is related to genetic alterations in precancerous solar keratosis (SK) lesions. Lintell et al. (2007) analyzed CNVs in genes associated with tumor development and cellular maintenance (SDHD and MMP12 genes) in the whole blood samples from affected SK and control cohort. It was found that 12 samples had CNVs in either the SDHD or MMP12 gene (amplified or deleted), with five of the samples exhibiting aberrations in both genes [40].

In contrast, the study of 85 primary melanomas arising from sun-protected (including palms, soles, and mucous membranes) and sun-exposed (including head, neck, trunk, and extremities) sites showed that CNVs in *GAB2* and *KIT* genes occur with the same frequency [41].

Frequency of single base substitutions (SBS) and CNVs in the exome sequences of spontaneous and neonatal UVB-induced mouse malignant melanoma (MM) have been studied. UV-induced MMs carried more SBSs than spontaneous MMs, but the levels of genomic instability, reflected by translocations and CNVs, were not different [42].

Shain et al. (2018) analyzed genomic and transcriptomic changes of melanoma from premalignant lesions (nevi, intermediate neoplasms, in situ melanomas, invasive melanomas) in 82 patients. Frequency of point mutations, resembling UV radiation-induced pattern, increased until melanoma invasion, at which point CNVs also became prevalent. The abundance of CNVs at the transition to invasive melanoma and thereafter was revealed [43]. 

Copy number alterations were also identified along with UV mutagenic signature, unexpectedly presented in acute lymphoblastic leukemia (ALL) samples [44]. However, ALL starts in the bone marrow, which is not available for UV radiation, hence it is impossible to assess definitely the relation of genetic changes to UV exposure. Overall, these studies indicate that CNVs in UV radiation exposed skin lesions may occur as a result of UV-induced genomic instability rather than via direct impact of UV radiation.

## 5. Chemical Mutagen-Induced CNVs In Vivo

The contribution of environmental pollution to CNV-related disease development poses a challenge to identify chemical agents that influence genomic instability. The CNVs include gains/losses of DNA segments, which can potentially disrupt regulation of gene expression by dosage or positional effects can affect the disease [45]. There is evidence of the input of CNVs, induced by chemicals in vivo, in development of animal and human diseases.

Genome regions in which the DNA copy number differed significantly between patients with asbestos-exposed and non-exposed lung tumors were detected for the first time using aCGH [46]. The aberrations were either low copy number gains or losses. The most significant altered regions were 2p21-p16.3, 5q35.3, 9q33.3-q34.11, 9q34.13-q34.3, 11p15.5, 14q11.2, and 19p13.1-p13.3. More gains than losses have been detected. Gain in chromosome regions 2p21-p23 was more frequent in the exposed group and have rarely been described in lung cancers. Interestingly, 12 genes that lie within the asbestos-related regions have previously been associated with fragile sites implicated in bladder cancer. Thus, first time copy number aberration profile related to asbestos exposure was obtained. 

Wikman et al. (2007) investigated a specific gene expression profile that correlates with the previously detected asbestos-associated CNVs [47]. By combining the gene expression data with the aCGH data, six distinct chromosomal regions that harbor both gene expression and CNVs were detected. The regions that were detected both in the expression and copy number data sets were considered prominently interesting. Asbestos-related areas were identified in 2p21-p16.3, 3p21.31, 5q35.2-q35.3, 16p13.3, 19p13.3-p13.1 and 22q12.3-q13.1. The largest significant region on chromosome 19p13.3–p13.1 showed a loss and down-regulation of genes in the exposed group and gain in the non-exposed patients. As a result, a combinatory profile of CNVs and expressional changes associated with asbestos exposure was obtained for lung carcinomas. One of the chromosomal regions affected by both CNVs and gene expression change in lung tumors of asbestos-exposed patients 2p21-p16.3 was evaluated as a possible marker for asbestos exposure. Analysis of 205 patients permitted to localize loss and allelic imbalances at 2p16.3-p16.2. Copy number loss at 2p16 is significantly associated with level of patients’ occupational exposure. Thus, the alterations at 2p16 combined with other markers could be useful in diagnosing asbestos-related lung cancer [48]. CNVs were analyzed in lung tumors from a population with chronic arsenic exposure, including lung squamous cell carcinomas (SqCCs) tumors from patients with no smoking history using aCGH. In tumors of patients exposed to arsenic recurrent deletions in 1q21.1, 7p22.3, 9q12, and 19q13.31 and gain at oncogene, candidates containing loci 19q13.33 were detected. The majority of 31 CNV-containing genes were clustered at two CNV hotspots at 19q distinctive for arsenic exposed group. The authors suggested that gain at 19q13.33 may activate oncogenes and drive genomic instability in lung tissue, increasing the risk of lung cancer development [49].

Postmortem brain samples of individuals with autism spectrum disorders were analyzed for developmental neurotoxicants polychlorinated biphenyls and polybrominated diphenyl ethers that bioaccumulate in lipid-rich tissues. The relationship between persistent organic pollutants levels in the human brain and dup(15) (q11q13), one of the most common CNVs observed in autism genetic diagnosis was found. Environmental factors leading to 15q11-q13 deletions or duplications have not been previously reported. Therefore, this exposure should be considered to be a potential environmental contributor of the CNVs [50]. However, the limitations of this study do not allow definitely stating whether the copy number alteration was specifically generated by the environmental chemical exposure.

There is evidence that CNVs can arise in response to therapy and more likely present adaptive genetic events for cancer cell growth [51]. Thus, short lasting drug treatment plans have been proposed to avoid adverse CNV-containing cell selection. 

CNVs of c-MYC before and after neoadjuvant chemotherapy with doxorubicin, cyclophophamid and docetaxel in different regimens in breast cancer patients were studied by FISH [52]. In the pre-neoadjuvant therapy group, c-MYC amplification (c-MYC copy number ≥6.0) was present in 15.1% while gains (c-MYC copy number ≥3.0) were identified in 53.8% cases. In post-neoadjuvan therapy group amplification of c-MYC was observed in 7.6%, while gains in 40.3% cases. In addition, in three samples, conversion from amplified to non-amplified status and in four samples conversion from non-amplified to amplified status (chemo-resistant group) was revealed. It was concluded that CNVs of c-MYC in chemotherapy-resistant tumors could occur during neoadjuvant chemotherapy. The authors suggest that evaluation of c-MYC CNVs after neoadjuvant chemotherapy can be used as a prognostic factor in patients with breast cancer.

Changes in CNVs were analyzed in women with breast cancer included in a randomized phase II trial evaluating the efficacy and safety of bevacizumab in combination with neoadjuvant treatment regimens [53]. Chemotherapy in combination with bevacizumab in non-responding tumors resulted in a frequency of gain > 30% at chromosomes 11q13.2 and 12p11.21 and > 30% deletion frequency was observed at chromosomes 6p21.33-p21.32, 8p, 11q13.5-q25, 13q31-q34, and 19q13. The good responders lose all aberrations during treatment and move towards a “normal” signal. The authors concluded that genetic heterogeneity has significant influence on the response to targeted treatment in combination with chemotherapy.

In four patients with colorectal cancer changes, CNVs have been analyzed by whole exome sequencing before and after neoadjuvant treatment with FOLFOX (leucovorin-modulated 5-fluorouracil and oxaliplatin combination). It was revealed that the CNVs persisted regardless of the administration of FOLFOX [54]. Taking into account the small size of the studied cohort it is not possible to make a comprehensive conclusion about impact of neoadjuvant treatment with FOLFOX on CNVs of patients with colorectal cancer. 

Kim et al. evaluated the contributions of CNVs exposure to air pollutants, and the interaction between the two on autism risk in children exposed from pregnancy through the second year of life [55]. This study showed that ozone, a major component of air pollution, may increase the risk of autism in children who have a high background level of copy number changes in their DNA. However, no correlation between air pollution exposure and the child’s CNV burden was detected. CNV information from the parents was not obtained. While this study established the importance of both genetic and environmental factors in autism, direct measures of their relative contributions and moreover, of the interactions between them are lacking. Thus, further work, in which de novo and inherited CNV are distinguished, is warranted. 

## 6. Chemical Mutagen- and Radiation-Induced CNVs In Vivo

Analysis of the consequences of radio-/chemotherapeutic treatment [56] and description of genetic signature of mice tumors generated by chemicals or radiation [57,58] allowed comparing the effects of radiation and chemical mutagens on CNVs.

CNV changes under chemo- and/or radiotherapy were described in non-small cell lung cancer patients [56]. In case of chemotherapy, patients received platinum plus gemcitabine, platinum plus vinorelbine, platinum plus taxanes, platinum plus pemetrexed. 21% observed coding mutations and 18% CNV were lost or gained during therapy. Mutational and CNV changes clustered in 16% and 8% patients, respectively. The type of radiochemotherapy but not the duration of therapy had impacted on the frequency of mutational changes.

CNVs in mouse thymic lymphomas induced by gamma irradiation and *N*-methyl-*N*-nitrosourea (MNU) were compared [57]. The copy number gains of chromosomes 4 and 5 were observed only in the radiation-induced lymphomas, and gains of chromosomes 10 and 14 were observed only in the MNU-induced lymphomas. Regional copy number losses in chromosomes 11, 16, and 19 frequently appeared in the radiation-induced lymphomas. In both groups, copy number gain of chromosome 15 was the most frequent event. 

Genomic analysis across murine soft-tissue sarcomas induced by 3-methylcholanthrene (MCA), oncogenic mutations (KrasG12D activation and p53 deletion), or ionizing radiation was performed. IR-induced p53 wild-type sarcomas exhibited higher number of genes affected by CNVs compared to MCA-induced p53 wild-type sarcomas. This trend was consistent for both copy number gains and losses. Moreover, sarcomas induced by MCA in p53 wild-type mice showed a lower number of genes affected by CNVs compared to sarcomas initiated by MCA and p53 loss. Thus, both ionizing radiation and p53 loss contribute to increase the number of CNVs during sarcomagenesis. Prevalence of losses over gains in radiation and MCA treated groups was observed [58].

Interestingly, the number of radiation-induced gains in vivo prevails over losses while gains and losses induced by chemical mutagens are found approximately in equal cases (Table 2). The difference in results obtained in vitro could be due to specificities of pharmacokinetics and pharmacodynamics of chemical compounds. In case of radiation-induced CNVs, the sensitivity of different organs to radiation should be considered.

## 7. Mutagen-Induced CNV in Germline

Germ cells mutations may occur as a result of errors in DNA replication during gametogenesis, inefficient repair of DNA damages or exposure to mutagens during lifetime [61]. Yet we still know little about the role of environmental agents in the etiology of heritable mutations because germ cell mutations are rare (10^−8^ per nucleotide) and difficult to detect because of technological limitations [60]. Many rodent germ cell mutagens have been identified; however, there are no accepted human germ cell mutagens [62]. Advances in NGS and aCGH have allowed the analysis of de novo mutations in germ cells at genome scale [18,59,63,64] and open up promising prospects for the development of this area. Germline CNVs are considered to be an important form of human genetic polymorphisms [63] and perfect marker for germline mutation study [65].

Adewoye et al. presented the results of the first genome-wide systematic study of germline mutations induced in laboratory mice after parental exposure to ionizing radiation [18]. The frequency of de novo CNV was investigated along with insertion/deletion events (indels) and single-nucleotide variants (SNV) in the offspring of irradiated males. The frequency of de novo CNV is significantly elevated in offspring of exposed fathers. Among the 14 unique germline CNV mutations found in the offspring of irradiated males, 12 were deletions and the other two were duplications. 

The effects of HU on germline CNVs was studied in mice [16] to test the idea that HU-induced replication stress will act as a CNV-inducing mutagen in germline mitotic divisions in the same manner as in cultured somatic cells [11]. However, HU did not increase the control level of CNV, perhaps due to highly toxic effects on sperm development or experimental variables related to HU pharmacology in mice. The authors suggest that the results obtained may also reflect different cellular responses to HU by cells in vitro and those in vivo.

A genome-wide CNV and DNA methylation analysis was realized in three generations of fungicide vinclozolin-treated mice. The vinclozolin was administered to pregnant mice. F1 generation sperm did not appear to have a significant increase in CNV mutations. In contrast, in F3 generation sperm were revealed mutated CNV sites [59]. The authors suggest that an environmental factor can promote transgenerational germline CNVs and epimutations in later generations.

The consequences of paternal exposure to benzo(a)pyrene (BaP), a ubiquitous environmental pollutant, on the mouse germline was studied using NGS and aCGH [60]. Paternal exposure to BaP induces single-nucleotide variants, indels and CNV in the offspring. There was an increase in duplication CNVs in the treated group and no evidence for induction of deletions. The similarity in the mutation spectra observed in the sperm of exposed males and their offspring supports the heritability of benzo(a)pyrene induced germ cell mutations. 

The only study of CNV in human germ cells was conducted in nine Vietnamese veterans with elevated concentrations of dioxin in their sera, together with their spouses and children [66]. It was shown that dioxin exposure might affect father’s genomes leading to de novo mutations in their children. De novo germline mutations, including SNV, short indels, CNV, or structural variation were identified by whole-genome sequencing. Out of eleven studied trios only in one case was de novo CNV (174.7 kb deletion on chromosome 15) identified in the offspring. Considering the limited number of studied cohort and low frequency of CNVs in the offspring of exposed people the impact of dioxin exposure on germline CNVs is still questionable.

## 8. Mutation Breeding and CNV

CNVs affect the phenotype of plants [3] and animals [4,67] since they can have a pronounced effect on gene expression and function. Mutation breeding is based on the induction of genetic variations; hence knowledge of the frequency and type of induced mutations is important for implementation of a mutation breeding program [68]. 

Analysis of the genome-wide structural consequences of chemical mutagen- or radiation-induced mutations in plants and animals obtained during selection works allows expanding understanding of mutagen-induced CNVs. In the reviews of breeding studies not only mutagen-induced CNVs but also indels in poplar [69] and fish [70] were included because of the few available examples, given that these genomic repeats differ only in size and can also characterize CNV mutagenesis. 

Fast neutron radiation has been used as a mutagen to develop extensive plant mutant collections. Genome-wide structural variants were observed among soybean plants sampled from a large fast neutron-exposed population. The prevalence of segmental duplications in addition to deletions within fast neutron-irradiated mutants was described. Certain deletions or duplications have been associated with quantitative changes in seed composition and short petiole mutant phenotype. Overall, this study demonstrates the utility of fast neutron-irradiated mutants as a source of novel genetic losses and gains [71].

aCGH platform was developed and applied for characterization of fast neutron bombardment-induced mutants in Medicago truncatula known as a model legume species [72]. Analysis of one of the mutants FN6191, which exhibited a hyper-nodulation phenotype, revealed a deletion region of 22 kb size on chromosome 4, encompassing the SUNN gene. Application of this platform allowed generating a database of CNV associated with Medicago truncatula mutant lines [73].

Gamma radiation mutagenesis has been used for decades to create populations of mutants in many crop species. Interspecific poplar hybrids, using gamma-irradiated pollen from Populus nigra to pollinate Populus deltoides were obtained. Detected mutations in the form of large-scale insertions and deletions cover the entire genome multiple times, with an average of 10 indels per gene. The size of indels ranged from whole chromosomes to small fragments [69].

Whole-genome sequencing was performed to reveal mutations in rice Red-1 derived from gamma-irradiated Oryza sativa L. ssp. Indica after several generations of selection [74]. Compared with the Oryza sativa, 9.19% of Red-1 genome sequence was altered. Among these alterations, there were 381,403 SNPs, 50,116 1–5 bp indels, 1,279 CNVs, and 10,026 presence/absence variations. It was shown that genes that are associated with cell components, binding function, catalytic activity and metabolic processes were susceptible to γ-radiation. The results obtained provide novel insights into the mechanisms by which radiation improves the beneficial properties in rice Red-1.

In the next study, the sequencing of whole genomes of rice established after several generations from gamma-irradiated Oryza sativa revealed single base substitution, short indel mutations, structural variations and CNVs. The mutations were scattered in all genomic regions across 12 rice chromosomes without apparent hotspots [68]. 

Two rice mutants GA-III-189 and GA-III-1052 with the dwarf phenotype, isolated among the descendants of gamma-irradiated cultivar Dongan (O. sativa L. japonica) were subjected to aCGH analysis [75]. Most of the CNVs identified were less than 10 kb in length. Ninety amplified and 18 deleted regions in GA-III-189, and 99 amplified and 11 deleted regions in GA-III-1052, were detected. Please note that CNVs were located on chromosome 12 in both GA-III-189 and -1052, which contained 39 commonly amplified regions in 29 genes. Integration of aCGH and gene expression data identified copy number aberrations and novel genes potentially involved in the dwarf phenotype.

SNVs, indels and CNVs were identified in genome of ethyl methanesulfonate (EMS)- and gamma irradiation-treated tomato Micro-Tom by a whole-genome sequencing. Mutations induced by EMS and gamma-ray irradiation occurred randomly throughout the genome. In addition, CNVs were found that were probably induced by mutagenesis in chromosome 6 of gamma irradiation-induced mutant line TOMJPG1269_1 and in chromosome 1 of EMS-induced mutant line EMS bulk set C_2. Subsequent PCR and sequencing analyses confirmed the deletion of a 37,390-bp fragment in the PCR product of TOMJPG1269_ 1. The deleted fragment consisted of four genes [76]. 

Mutagenesis is one of the approaches to introduce genetic diversity in bananas. Officially released mutant banana varieties were created using gamma rays. In the study by Datta et al. a low-coverage whole-genome sequencing approach was applied in bananas to recover copy number caused by treatment with gamma irradiation [77]. The genome of commercially released mutant cultivar ‘Novaria’ contains multiple predicted large deletions, ranging from 0.3 to 3.8 Mbp spanning 189 coding regions. Then a pipeline for gamma irradiation mutagenesis and screening for CNV in Cavendish bananas was developed using the cultivar ‘Williams’. Mutations were recovered in 70% of lines treated with 20 Gy and 60% of the lines treated with 40 Gy. While deletion events predominate, insertions were identified in 20 Gy-treated material. 

The first successful mutagenesis in a marine aquaculture fish species, Japanese flounder, Paralichthys olivaceus, was realized for breeding using a novel atmosphere and room temperature plasma (ARTP) mutagenesis tool [69]. ARTP treatment was applied for the fertilized eggs and sperm. Whole-genome sequencing revealed 240,722 to 322,978 SNPs and 82,149 to 86,798 indels. The authors concluded that ARTP mutagenesis is a useful method for the breeding of fish species. 

## 9. Mutagen-Induced CNVs in Adaptive Evolution

A study of experimental evolution in Caenorhabditis elegans over more than 200 generations revealed a rapid increase in frequency of gene copy number variants and demonstrated great potential of CNV for evolutionary adaptation [78]. However, the role of CNV in adaptive evolution remains mostly unexplained and little is known about the extent to which environmental stressors accelerate mutation rates and influence evolution. Moreover, most studies on stressors have focused on unicellular organisms and point mutations rather than large-scale deletions and duplications (CNVs) [79].

It was earlier suggested that CNVs can arise through replication defects [10]. Hull et al. proposed a general mechanism for environmentally stimulated CNV and validate this mechanism for the development of copper resistance in budding yeast [80]. Transcription can restrict replication fork progression and stability, leading to increased CNV mutation rates at transcribed loci. Thus, environmental factors may promote CNV formation in the loci whose transcription they can stimulate. Consequently, mutations can occur in the genes responsible for adaptation to the current environment. Yeast cells exposed to copper stimulate copy number amplification of the actively transcribed copper resistance gene CUP1, leading to the rapid rise of adapted clones. Thus, stimulated CNV provides cells with a remarkable and unexpected ability to alter their own genome in response to the environment. At the same time, the authors rightly pointed out that CNV mutations at highly expressed genes may cause deleterious CNV in housekeeping genes. Stimulated CNV is therefore an imperfect but useful mechanism to develop adaptations. 

CNV mutations were estimated in *Daphnia pulex* in the presence of ecologically relevant concentrations of copper and nickel. Sequencing of *Daphnia pulex* lines exposed to copper, nickel or to a mixture of nickel and copper after an average of 103 generations revealed significantly enhanced rates of deletions and duplications. The presence of nickel and copper increased deletion and duplication rates fourfold. A total of 916 de novo single-nucleotide mutations and small (1-50 bp) indels, as well as 776 deletions and 406 duplications larger than 500bp were detected [79].

## 10. Identification of CNV-Inducing Mutagens Through the Mechanisms of CNV Formation

One possible way to identify CNV-inducing mutagens is a clear understanding of the underlying mechanisms by which CNVs are formed [10,81]. Several publications elucidate the mechanisms of CNV formation in experiments with application of CNV-inducing mutagens.

To identify and characterize compounds that may induce de novo CNVs in humans it is critically important to consider the existence of both nonrecurrent and recurrent CNVs. Nonrecurrent de novo CNVs originate primarily in mitotic cells through replication-dependent DNA repair pathways. Recurrent de novo CNVs are most often formed in meiotic cells through homologous recombination between nonallelic large low copy repeats. Different mechanisms of de novo copy number mutagenesis suggest the existence of different classes of CNV-inducing environmental mutagens [82].

It was shown that agents that perturb normal replication and create conditions of replication stress, including aphidicolin [22,27], HU [23] and aflatoxin B1 [28] are potent inducers of non-recurrent CNVs in cultured human cells. Resemblance of sizes of aphidicolin- and HU-induced CNVs to spontaneously arising ones during cell cultivation proves that there are genomic loci prone to occurrence of de novo CNVs due to errors in replication (e.g., replication fork stalling) [10].

To test whether the non-homologous end joining (NHEJ) DNA repair pathway is involved in the formation of non-recurrent CNVs, the effects of loss of gene Xrcc4, responsible for the NHEJ in mouse embryonic stem cells following treatment with low doses of aphidicolin were studied. Cells lacking NHEJ displayed unaltered CNV frequencies, locations, and breakpoint structures compared to normal cells. These results establish that CNV mutations arise by a mutagenic mechanism other than canonical NHEJ [83].

To better understand the role of DNA replication in CNV mutation across different species, the statistic R, as measure of dynamic replication fork progression, was applied in the human, mouse and Drosophila genomes. A bioinformatics analysis was conducted on the basis of high-resolution data of DNA replication timing in species studied. Correlation of a reduced replication rate with increased genome instability at CNV loci was revealed. The previously described involvement of DNA replicative mechanisms in CNV formation [83] was confirmed on the base of DNA replication dynamics data in different species [84].

The potential link between CNVs and common fragile sites (CFSs) seen as breaks or gaps on metaphase chromosomes was studied in several experimental cell systems by Wilson et al. [85]. CNV hotspots and CFSs occurred at the same human loci and corresponded to the largest active transcription units in human fibroblast and mouse embryonic stem cells. These results indicate that active large transcription units drive extreme locus-specific genomic instability under replication stress, resulting in both CNVs and CFSs.

## 11. Conclusions

There is ongoing concern about the consequences of mutations in humans and biota arising from environmental exposure. Genomic instability in somatic cells can lead to cancer in the affected individuals. In contrast, germ cell mutations can affect future generations, and also lead to reduction in fitness in population, reproduction impairment, higher rates of offspring carrying deleterious mutations, and population decline [86].

Presently, monitoring of genomic stability is based on endpoints, such as DNA single- and double-strand breaks, point mutations, chromosomal aberrations, micronuclei both in vitro and in vivo. In the last decade, considerable activities have been carried out worldwide with the aim of optimizing strategies for genotoxicity testing, both with respect to the basic in vitro testing battery and to in vivo follow-up tests [87].

The results of studies presented in this review definitely indicate the ability of environmental mutagens to influence CNV in humans, animals and plants. Additional studies will be needed with other model systems and other mutagens to investigate and further understand the influence of chemical and radiation exposure on generating CNVs. Various effects of radiation and chemical mutagens in vitro and in vivo on CNVs certainly requires further elucidation in order to explain their localization in genome and prevalence of gains over losses in some loci and the opposite effect in others.

Crucially, standardized approaches should be developed to discriminate (potentially) deleterious and biologically irrelevant CNVs. Obviously, in the near future, CNVs can be included as a new endpoint in the battery of mutagenicity tests. Such studies could contribute to a more detailed description of the spectrum of genetic damage induced by environmental mutagens.

## Figures and Tables

**Table 1:** Summary of studies on induction of copy number variations (CNVs) using radiation and chemical mutagens in vitro with selected examples of deletion and duplication loci. NA = not applicable.

Mutagen	Cells	Chromosomes Most Frequently Involved in Gains	Chromosomes Most Frequently Involved in Losses	Prevailing CNV in the Genome	Reference
γ-radiation	Human lymphoblastoid cell line TK6	3q, 13q and 20q	X	Gain	[19]
X-ray	Human lung cancer cell line A549	3 and 5	X, 3 and 5	Loss	[20]
X-ray	Human immortalized fibroblasts hTERT	6p, 7q11.22, 9p and 16q23.1	3q13.31 and 15q	Gain	[21]
X-ray	Primary human fibroblasts	1q44 and 10q26.11	3p14.2, 7q11.2 and 7p12.1-7q11.1	Loss	[24]
Accelerated electrons	Human blood leukocytes	7q11.22 and 9q21.3	NA	Gain	[25]
Aphidicolin	Normal human fibroblasts HGMDFN090	1q44, 7q21.11-q31.3, 10q25.2 and 15q22.2	3q26.3, 3q13.31, 10q11.23-q21.1 13q31.3 and 15q22.2	Loss	[22]
Aphidicolin	Human immortalized fibroblasts hTERT	1, 9, 10 and 16	3, 7, 11, 13, 15 and 18	Loss	[26]
Hydroxyurea	Human immortalized fibroblasts hTERT	1p36.31- 1q25.1 and 3p25.3- 3q27.3	3q13.31 and 7q11.22-7q33	Loss	[23]
EMS	Zebrafish fibroblast cell line	13 and 20	5, 8 and 18	Loss	[27]
Cytosine arabinoside	Zebrafish fibroblast cell line	5	NA	Gain	[27]
Aflatoxin B1	Human blood leukocytes	NA	8p21.2 and 15q11.2	Loss	[28]

**Table 2:** Summary of studies on induction of CNVs using radiation and chemical mutagens in vivo with selected examples of deletion and duplication loci. NA = not applicable.

Mutagen	Organism and cells	Chromosomes/Genes Most Frequently Involved in Gains	Chromosomes/Genes Most Frequently Involved in Losses	Prevailing CNV in the Genome	Reference
γ-radiation	Rat mammary carcinoma	1q12	3q35-q36, 5q32, and 7q11	Loss	[29]
γ-radiation	NF1 mutant mouse models of secondary malignant neoplasm	1	11	Loss	[17]
γ-radiation	Wild-type mouse models of secondary malignant neoplasm	1	5 and 11	Gain	[17]
Radium-226	Human breast cancer	2q, 4, 17, 21q, and 22q	6q	Gain	[30]
Ionizing radiation during Chernobyl accident	Human papillary thyroid carcinoma	1p34, 1q21, 2p11.1-pter, 2q11.2-13, 3q26.2-26.3, 5q23-31, 6p21.3-pter, 7q11, 9q13-q33, 12q22-qter, 13q32-qter, 17q11.1-qter, 19, 20, and X	1q42, 13q21, and 15q11.1-14	Gain	[32]
Ionizing radiation during Chernobyl accident	Human childhood thyroid tumors	2, 7q11.2-21, 13q21-22, and 21	16p, 16q, 20q, and 22q	Gain	[33]
Ionizing radiation during Chernobyl accident	Human childhood thyroid tumors	19 and 21	1p36.31-35.3, 1p33-cen, 1q23.2-32.1, 6, 9p21.1-pter, 9q31.1, 9p23-q33.2, and 13	Loss	[35]
Ionizing radiation during Chernobyl accident	Human childhood thyroid tumors	1p, 5p, 9q, 12q, 13q, 16p, 21q, and 22q	1q, 6q, 9q, 10q, 13q, 14q, 21q, and 22q	NA	[36]
Ionizing radiation during Chernobyl accident	Human breast cancer	7q11.22-11.23, 7q21.3, 16q24.3, 17q21.31, and 20p11.23-11.21	1p21.1, 2q35, and 6p22.2	Gain	[37]
A-bomb	Human breast cancer	c-Myc, HER2	NA	Gain	[38]
Possibly solar UV radiation	Human whole blood	MMP12	SDHD	Gain	[40]
Possibly solar UV radiation	Human primary melanoma	GAB2, KIT	NA	Gain	[41]
UV radiation	Mouse malignant melanoma	NA	Trp63, Pten	Loss	[42]
Possibly solar UV radiation	Human melanocytic neoplasms	BRAF, NRAS, and MAP2K1	NA	NA	[43]
Asbestos	Human lung cancer	2p21-p23	9p23-pter	Gain	[46]
Asbestos	Human lung cancer	2p21–p16.3	3p21.31, 5q35.2–q35.3, and 19pl3.3–p13.1	Loss	[47]
Asbestos	Human lung cancer	NA	2p16	Loss	[48]
Arsenic	Human lung cancer	19q13.33	1q21.1, 7p22.3, 9q12, and 19q13.31	Loss	[49]
Polychlorinated biphenyls and polybrominated diphenyl ethers	Human brain samples	15q11q13	NA	Gain	[50]
Combination of doxorubicin, cyclophophamid and docetaxel	Human breast cancer	c-Myc	NA	Gain	[52]
Bevacizumab plus neoadjuvant chemotherapy	Human breast cancer	11q13.2 and 12p11.21	6p21.33-p21.3, 8p, 11q13.5-q25, 13q31-q34, and 19q13	Loss	[53]
FOLFOX	Human colorectal cancer	7q21, 10q22, and 10q23	NA	NA	54
Ozone in the air	Blood from patients with autism	NA	NA	Gain	[55]
Radio- and chemotherapy	Human lung cancer	FGFR1	CDKN2A	Gain	[56]
γ-radiation	Mouse thymic lymphoma	4, 5, and 15	11, 16, and 19	NA	[57]
N-methyl-N-nitrosourea	Mouse thymic lymphoma	10, 14, and 15	NA	NA	[57]
γ-radiation	Murine soft-tissue sarcoma	NA	NA	Loss	[58]
MCA	Murine soft-tissue sarcoma	NA	NA	Loss	[58]
Vinclozolin	Murine germ cells	2 and 9	NA	Gain	[59]
Benzo(a)pyrene	Murine germ cells	3, 5 and 16	3	Gain	[60]

## Abbreviations

A-bomb	Atomic bomb
ARTP	Atmosphere and room temperature plasma
Ara-C	Cytosine arabinoside
CFSs	Common fragile sites
CNV	Copy number variation
aCGH	Array comparative genomic hybridization
NGS	Next-generation sequencing
EMS	Ethyl methanesulfonate
FRA3B	Fragile Site Aphidicolin Type, Fra(3)
FRA16D	Fragile Site Aphidicolin Type, Fra(16)
FOLFOX	Leucovorin-modulated 5-fluorouracil and oxaliplatin combination)
HU	Hydroxyurea
IR	Ionizing radiation
MNU	N-methyl-N-nitrosourea
MCA	3-methylcholanthrene
NHEJ	Non-homologous end joining
POD-FISH	Parental origin determination fluorescence in situ hybridization
PTC	Papillary thyroid carcinoma
SqCCs	Lung squamous cell carcinomas
SNV	Single-nucleotide variants
